# Meta‐analysis of the use of sterilized mosquito net mesh for inguinal hernia repair in less economically developed countries

**DOI:** 10.1002/bjs5.50147

**Published:** 2019-02-27

**Authors:** M. H. Ahmad, S. Pathak, K. D. Clement, E. H. Aly

**Affiliations:** ^1^ University Hospitals of Leicester Leicester UK; ^2^ Queen Elizabeth University Hospital Glasgow UK; ^3^ Department of General Surgery, Aberdeen Royal Infirmary Aberdeen UK; ^4^ University of Aberdeen Aberdeen UK

## Abstract

**Background:**

Inguinal hernias are common in less economically developed countries (LEDCs), and associated with significant morbidity and mortality. Tension‐free mesh repair is the standard treatment worldwide. Lack of resources combined with the high cost of commercial synthetic mesh (CSM) have limited its use in LEDCs. Sterilized mosquito net mesh (MNM) has emerged as a low‐cost, readily available alternative to CSM. The aim of this systematic review and meta‐analysis was to evaluate the safety and efficacy of MNM for the use in hernia repair in LEDCs.

**Methods:**

A systematic review and data meta‐analysis of all published articles from inception to August 2018 was performed. Cochrane Central Register of Controlled Trials, MEDLINE and Embase databases were searched. The primary outcome measure was the overall postoperative complication rate of hernia repair when using MNM. Secondary outcome measures were comparisons between MNM and CSM with regard to overall complication rate, wound infection, chronic pain and haematoma formation.

**Results:**

A total of nine studies were considered relevant (3 RCTs, 1 non‐randomized trial and 5 prospective studies), providing a total cohort of 1085 patients using MNM. The overall complication rate for hernia repair using MNM was 9·3 per cent. There was no significant difference between MNM and CSM regarding the overall postoperative complication rate (odds ratio 0·99, 95 per cent c.i. 0·65 to 1·53; *P* = 0·98), severe or chronic pain (OR 2·52, 0·36 to 17·42; *P* = 0·35), infection (OR 0·56, 0·19 to 1·61; *P* = 0·28) or haematoma (OR 1·05, 0·62 to 1·78; *P* = 0·86).

**Conclusion:**

MNM has a low overall postoperative complication rate and is unlikely to be inferior to CSM in terms of safety and efficacy. MNM is a suitable low‐cost alternative to CSM in the presence of financial constraint.

## Introduction

Hernia repair is one of the most commonly performed surgical operations worldwide^1^. Less economically developed countries (LEDCs) often have a significant number of people with inguinal hernia, with an estimated prevalence of 3·2 (range 2·8–3·5) per cent in Ghana^2^. The incidence of symptomatic hernia in Africa is reported to be approximately 200 per 100000 people^3^, and in Tanzania the incidence is as high as 5·4 per cent^4^. In both studies^2^, ^4^ the prevalence of inguinal hernia was compared against the current rate of hernia repair, and it was determined that there would be a backlog of approximately 1 million patients over a 10‐year period. This significant burden of surgical work has been recognized for more than 30 years^5^. Strangulated inguinal hernia has been reported to have a mortality rate of as high as 40 per cent in low‐income countries^6^. The prevalence of symptomatic hernia may be associated with delayed repair, and thus higher morbidity and mortality. Beyond this, poor infrastructure, shortage of medical facilities and low numbers of surgeons per population are issues faced by many LEDCs^7^.

In the mid 1980s, Lichtenstein tension‐free repair was advocated to deal with inguinal hernia in a way that avoided reliance on sutures and associated suture tension^8^, ^9^. Lichtenstein hernia repair with a commercial synthetic mesh (CSM) is now the most common technique used in the developed world^10^. A recent Cochrane review^11^ concluded that the use of mesh was associated with fewer recurrences, a shorter duration of surgery and a shorter length of hospital stay compared with non‐mesh repair. However, the cost of CSM has remained prohibitive in LEDCs^12^. Sterilized mosquito net mesh (MNM) may be a low‐cost alternative in these countries as an easily accessible substitute^13^.

The cost of importing CSM to low‐income countries is commonly reported to be approximately €90^13^, ^14^. This is in stark contrast to MNM, which costs consistently less than €1 for the material itself^13^, ^14^, ^15^. Following this, the mesh must be sterilized and packaged. Sterilization is most commonly carried out using an autoclave, which is cost‐effective and used widely throughout sub‐Saharan Africa^16^. Even taking into consideration the cost of local sterilization and packing, MNM remains considerably cheaper than CSM.

CSM is commonly made from polypropylene, which is easily sterilized. MNM is usually made from a number of materials, including a co‐polymer of polyethylene and polypropylene^14^ in varying proportions, reflecting manufacturer and country of origin. This variation can impact on the material properties of the mesh, related to the method of sterilization used^17^.

The aims of the presented systematic review and meta‐analysis were to evaluate current evidence investigating the safety and efficacy of MNM for groin hernia in LEDCs. The primary outcome measure was the overall postoperative complication rate of hernia repair using MNM. Secondary outcome measures were comparisons between MNM and CSM with regard to overall complication rates, wound infection, chronic pain and haematoma formation.

## Methods

**Figure 1:**
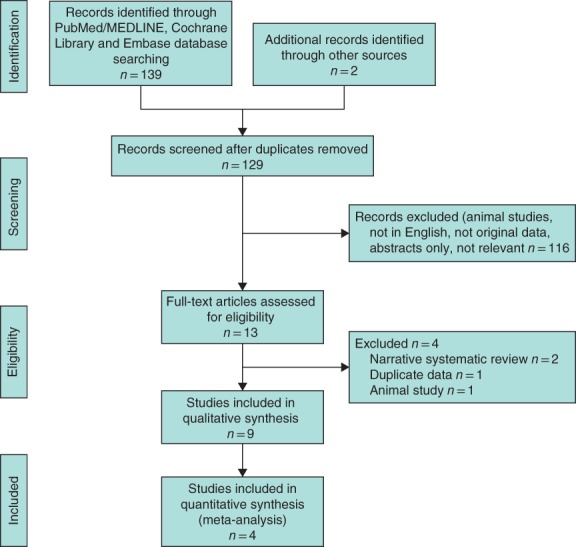
PRISMA diagram for the review

This systematic review and meta‐analysis was conducted according to a predefined protocol and in accordance with the principles recommended in the PRISMA guidelines^18^ (*Fig*. 1).

Eligible studies were included provided they met the following criteria: RCT, non‐randomized controlled trial or observational study relating to clinical studies on humans involving NMN for hernia repair, either in isolation or in comparison with CSM. Only original studies containing original data were included.

Animal studies, non‐English‐language studies, studies for which only an abstract was available, non‐clinical studies, studies with duplicate data, and narrative reviews were all excluded.

### Data sources

Eligible studies were identified by performing searches in the Cochrane Central Register of Controlled Trials, PubMed/MEDLINE and Embase, as resources to identify relevant manuscripts. Search term key words were ‘hernia’, ‘mesh’, ‘mosquito mesh’ and ‘hernia repair’. Databases were searched from inception to August 2018. Reference lists of relevant studies were also inspected for inclusion.

Studies retrieved from the searches were reviewed independently by two authors. Those that met the inclusion criteria were shortlisted for data extraction. Disagreements were resolved by discussion with a third author when necessary.

### Data extraction

Two reviewers extracted the data from shortlisted studies on study type, setting, country, population, sample size, mesh type, cost, sterilization techniques, type of surgery, study population demographics, baseline characteristics, intervention and postoperative complications. Data were recorded in a standard form, and risk‐of‐bias assessments were completed contemporaneously.

The primary outcome measure was the overall postoperative complication rate of hernia repair using MNM, including haematoma, graft rejection, infection and pain. Secondary outcome measures were comparisons between MNM and CSM with regard to overall complication rates, infection, chronic pain and haematoma formation.

### Risk of bias

Risk of bias was assessed independently by two authors using the Cochrane risk‐of‐bias checklist as published in the Cochrane Handbook for Systematic Reviews of Interventions version 5.1.0^19^. This evaluated random sequence generation and allocation concealment (selection bias), blinding of participants and personnel (performance bias), blinding of outcome assessment (detection bias), incomplete outcome data (attrition bias), selective reporting (reporting bias), and other sources of bias. Each of these domains was assessed to be at low, medium or high risk of bias.

### Statistical analysis and data synthesis

Statistical analysis and data synthesis was conducted using Review Manager version 5.3.5 (The Cochrane Collaboration, The Nordic Cochrane Centre, Copenhagen, Denmark). Outcomes deemed relevant from the included studies were assessed for estimation of treatment effects. Odds ratios (ORs) and 95 per cent c.i. were calculated for dichotomous outcomes. The mean difference with 95 per cent c.i. was calculated for continuous outcomes. Meta‐analyses were assessed for heterogeneity using the χ^2^ test and *I* ^2^ statistic (considered significant if the χ^2^ statistic had a *P* value of less than 0·100, or *I* ^2^ was greater than 50 per cent). Where heterogeneity was found to be significant, analyses were carried out using a random‐effects model. For non‐randomized trials, data were represented numerically and a cumulative analysis was performed. Where possible, statistical analysis and data synthesis were limited to studies reporting intention‐to‐treat protocols. Where data were missing, information was sought from the study authors. If this information remained unavailable, outcomes were inferred from existing data using statistical methods. No subgroup or metaregression analysis was performed.

## Results

### Description of studies

A total of 139 records were found through database searching. Following application of data exclusion criteria, nine studies were finally included (3 RCTs^13^, ^20^, ^21^, 1 non‐RCT^14^ and 5 studies using prospectively developed databases^22^, ^23^, ^24^, ^25^, ^26^). All studies took place in LEDCs, two in the Indian subcontinent and seven in Africa. All four controlled trials^13^, ^14^, ^20^, ^21^ compared MNM with CSM; the remaining studies focused solely on MNM.

### Participants

Data from the nine studies^13^, ^14^, ^20^, ^21^, ^22^, ^23^, ^24^, ^25^, ^26^ were extracted to form an aggregate quantitative synthesis including a total cohort of 1360 patients (1085 patients in the MNM intervention and 275 in the CSM control group). The mean age of participants in the studies ranged from 33 to 52 years, and the mean length of follow‐up varied from 1 month to 5 years (*Tables* *S1* and *S2*, supporting information).

### Assessment of bias

Risk‐of‐bias assessments were conducted for the four comparative studies that underwent meta‐analysis (*Table* 1). Two^20^, ^21^ were found to be at low risk of bias and two^13^, ^14^ had a high risk of selection and attrition bias.

**Table 1:** Risk‐of‐bias summary of individual RCTs and non‐randomized controlled trial assessed by Cochrane risk‐of‐bias checklist

	Random sequence generation (selection bias)	Allocation concealment (selection bias)	Blinding of participants and personnel (performance bias)	Blinding of outcome assessment (detection bias)	Incomplete outcome data (attrition bias)	Selective reporting (reporting bias)	Other bias
Chauhan *et al*.^21 ^	+	+	+	+	+	+	+
Freudenberg *et al*.^13 ^	?	+	+	+	?	+	+
Löfgren *et al*.^20 ^	+	+	+	+	+	+	+
Tongaonkar *et al*.^14 ^	−	−	−	+	?	+	?

+, Low risk of bias; ?, unclear risk of bias; −, high risk of bias.

### Primary outcome

**Figure 2:**
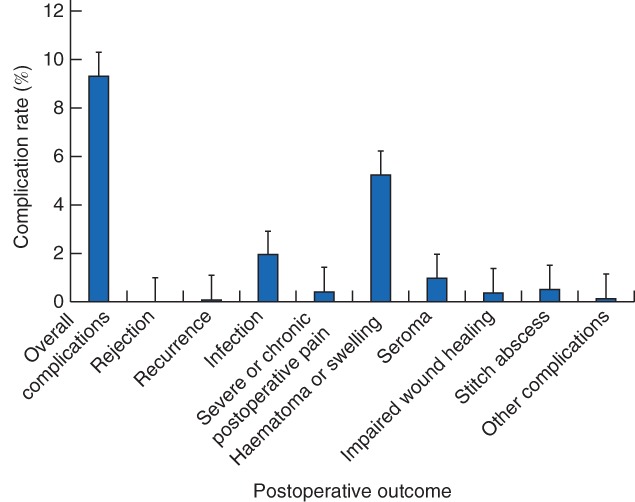
Postoperative outcomes for hernia repair using mosquito net mesh. There were 1085 hernias in total. Error bars denote 95 per cent confidence intervals

The overall postoperative complication rate was 9·3 per cent, with haematoma formation or swelling accounting for more than half of all complications. The incidence of graft rejection was 0 per cent, infection 1·9 per cent and impaired wound healing 0·4 per cent (*Fig*. 2). Individual postoperative outcomes for each study are summarized in *Table* *S3* (supporting information).

### Secondary outcomes

Owing to the limited number of RCTs, the single non‐RCT was included in the meta‐analysis. Four studies (3 RCTs, 1 non‐RCT)^13^, ^14^, ^20^, ^21^ compared MNM and CSM with regard to overall complication rate, severe or chronic postoperative pain, postoperative infection and haematoma formation. A random‐effects meta‐analysis did not demonstrate a statistically significant difference between the two groups.

**Figure 3:**
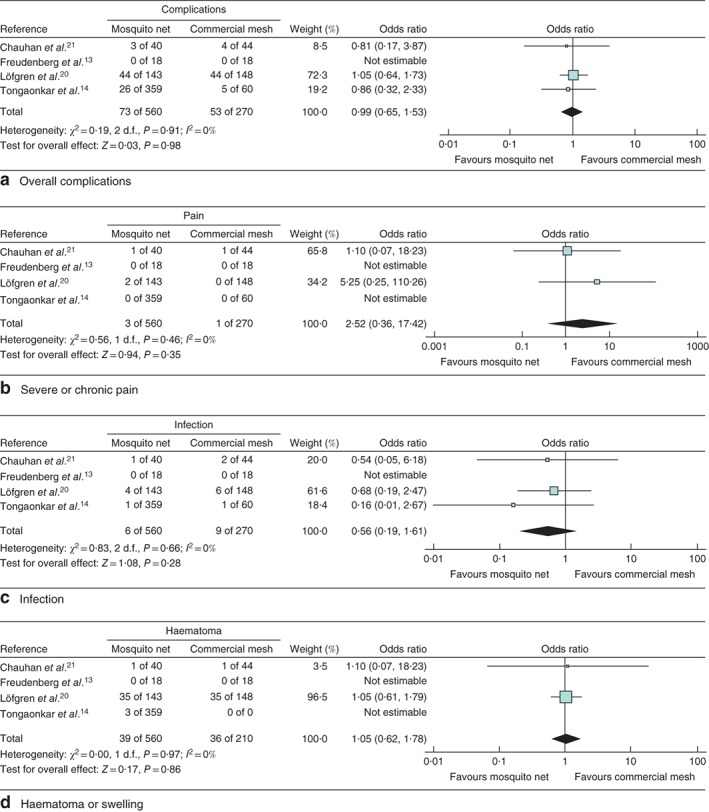
Forest plots comparing complications and pain after hernia repair with mosquito net *versus* commercial mesh. **a** Overall complications, **b** severe or chronic pain, **c** infection and **d** haematoma or swelling. A Mantel–Haenszel fixed‐effect model was used for meta‐analysis. Odds ratios are shown with 95 per cent confidence intervals

The overall complication rate revealed a pooled OR of 0·99 (95 per cent c.i. 0·65 to 1·53; *P* = 0·98), with no evidence of heterogeneity (*I* ^2^ = 0 per cent, *P* = 0·91) (*Fig*. 3 *a*). For severe or chronic pain, the pooled OR was 2·52 (0·36 to 17·42; *P* = 0·35), with no heterogeneity (*I* ^2^ = 0 per cent, *P* = 0·46) (*Fig*. 3 *b*). For infection the pooled OR was 0·56 (0·19 to 1·61; *P* = 0·28), with no heterogeneity (*I* ^2^ = 0 per cent, *P* = 0·66) (*Fig*. 3 *c*), and for haematoma the pooled OR was 1·05 (0·62 to 1·78; *P* = 0·86), with no heterogeneity (*I* ^2^ = 0 per cent, *P* = 0·97) (*Fig*. 3 *d*).

## Discussion

The results of the present meta‐analysis support those of a previous review^27^ and confirm the overall low rate of complications using MNM. This meta‐analysis combined individual data from different international centres with an overall cohort of 1085 patients, and demonstrated that patients undergoing inguinal hernia repair with MNM had a low rate of postoperative complications, comparable to that with CSM. Analysis of secondary outcome measures was not able to demonstrate a statistically significant difference in overall complication rates, infection rates, incidence of chronic pain and haematoma formation between MNM and CSM.

Sterilized MNM is a low‐cost alternative to CSM and has the potential to overcome some of the barriers to prompt surgical treatment. Data in the individual RCTs demonstrated that MNM can cost as little as €0·02 for a 15 × 15‐cm strip that can be used in place of traditional CSM.

Issues surrounding the sterilization of MNM are important. Previous research^18^ has suggested that not all MNMs will be suitable for autoclave sterilization. One study^17^ showed that exposure of meshes to temperatures of 121 °C caused some meshes to shrink by 30–50 per cent, with degradation of physical material properties. Further work should aim to create a standard protocol for the sterilization of MNM with no detrimental impact on its material properties.

The main limitation of the present study was that it included all trials published from inception to August 2018. Change in clinical practice over this interval, as well as surgical innovation and population change in LEDCs, may be important. Design and methodological limitations in the studies included in the quantitative synthesis may also have influenced the results. One of the four studies in the meta‐analysis was non‐randomized, and this would have introduced a degree of both selection bias and information bias secondary to potential confounders^14^. Of the three RCTs included, one^13^ was deemed to have a high risk of selection and attrition bias. The remaining five studies^22^, ^23^, ^24^, ^25^, ^26^ were classified as prospective observational studies, all of which had significant design and methodological limitations.

The primary outcome measure of complication rates after mesh insertion may be less useful than the rate of hernia recurrence after repair, but this could not be considered owing to the lack of long‐term data provided. No study included long‐term follow‐up of patients, so long‐term safety and efficacy remain uncertain. Traditional CSMs undergo rigorous quality control testing, accounting for their significantly higher cost compared with MNM, but they are known to be durable. This highlights an important knowledge gap in the long‐term performance of MNM and demonstrates the need for a larger body of evidence before definitive recommendations can be made.

## Disclosure

The authors declare no conflict of interest.

## Supporting information


**Table S1** Study characteristics and mesh data
**Table S2** Study cohort distribution
**Table S3** Outcomes: post‐operative complications
