# Supplementary material for: Meta‐analysis of the use of sterilized mosquito net mesh for inguinal hernia repair in less economically developed countries
Source: BJS Open. 2019 Feb 27;3(4):429–35. doi: 10.1002/bjs5.50147 (PMC6681152; doi:10.1002/bjs5.50147)
Supplement: Supplementary file 1 — Table S1 Study characteristics and mesh data Table S2 Study cohort distribution Table S3 Outcomes: post‐operative complications [file BJS5-3-429-s001.docx]

**BJS5_50147**

**Meta-analysis of the use of sterilized mosquito net mesh for inguinal hernia repair in less economically developed countries**

**M. H. Ahmad, S. Pathak, K. D. Clement and E. H. Aly**

**Table S1** Study characteristics and mesh data

| **Author** | **Year** | **Journal** | **Study design/Level of evidence** | **Dates of study** | **Country** | **Mesh type** | **Mesh Material** | **Cost $US (and Size)** | **Sterilisation procedure for mosquito net** |
| --- | --- | --- | --- | --- | --- | --- | --- | --- | --- |
| Lofgren et al. | 2016 | NEJM | RCT, double blind | Feb 2012 - Oct 2013 | Eastern Uganda | Mosquito net mesh | 100% polyethylene | Less than $1, (38.0 g per square meter, 1.5mm pore size) | Autoclave (121°C) |
|  |  |  |  |  |  | Commercial | 100% polypropylene mesh | $125 (53.7g per square meter, 1.9mm pore size | - |
| Chauhan et al. | 2007 | World J. Surg | RCT | Jan 2005–2006 | India | Indigenous Mesh | Polypropylene | $13 (size not reported) | Not reported |
|  |  |  |  |  |  | Commercial | Prolene (polypropylene) | $178 (size not reported) | - |
| Freudenberg et al. | 2006 | World J. Surg | RCT, Clinical randomized double-blind study | Aug–Oct 2005 | Burkina-Faso | Mosquito net mesh | Nylon (Polyamide 6-6) | $0.0043 (10cm x 15cm) | Autoclave (temperature not reported) |
|  |  |  |  |  |  | Commercial | Ultrapro (polypropylene with polyglactin) | $108 (10cm x 15cm) | - |
| Tongaonkar et al. | 2003 | Indian J. Surg | Non-randomized controlled trial | May 1996 – Jan 2002 | India | Mosquito net mesh | Poly(ethylene–co-polypropylene) | $0.02, (15cm x 15cm) | Autoclave (132°C) |
|  |  |  |  |  |  | Commercial | Prolene & Marlex | $75 (15cm x 15cm) | - |
| Oribabor et al. | 2015 | Nigerian J. Surg | Prospective Study | Jan 2012 - Dec 2013 | Nigeria | Mosquito net mesh | Locally ­sourced, polymer not reported | (10cm Χ 8 cm) | Autoclave (temperature not reported) |
| Stephenson et al. | 2011 | World J. Surg | Prospective Study | 2011 | Ivory Coast | Mosquito net mesh | Copolymer (50% polypropylene and 50% polyethylene), from India | less than less than $2.15 (10cm x 12 cm) | Autoclave (121°C) |
| Clarke et al. | 2008 | Hernia | Prospective Study | Nov 5–9, 2007 | Ghana | Mosquito net mesh | 100% polyester, manufactured and donated by Scotmas Limited | $1.50 (7cm x 15cm) | Autoclave (134°C) |
| Yenli et al. | 2017 | Ghana Med J. | Prospective Study | Aug 2010 - Dec 2013 | Ghana | Mosquito net mesh | Polypropylene | $ 1.8 (GH₵7.2) | Autoclave (not reported) |
| Rouet et al. | 2018 | J Visc Surg | Prospective Study | Jan - Nov 2013 | Cameroon | Mosquito net mesh | Polyester | $0.21 | Autoclave |

**Table S2** Study cohort distribution

| **Author** | **Mesh type** | **No. of Hernias** | **Mean Age (years) ± SD** | **Follow-up Period** | **Scrotal Hernia (%)** | **Inguinal hernia (%)** | **Recurrent Hernia (%)** | **Incisional Hernia (%)** | **Other/ unclassified Hernia (%)** |
| --- | --- | --- | --- | --- | --- | --- | --- | --- | --- |
| Lofgren et al. | Mosquito net mesh | 151 | 45.1 ±17.6 | 1 year | 38.7 | 61.3 | - | - | - |
|  | Commercial | 151 | 46.4 ±17.8 | 1 year | 39.6 | 60.4 | - | - | - |
| Chauhan et al. | Indigenous Mesh | 40 | 46.98 | 13.73 ±3.047 months | - | 100 | - | - | - |
|  | Commercial | 44 | 44.18 | 13.0 ±3.570 months | - | 100 | - | - | - |
| Freudenberg et al. | Mosquito net mesh | 20 | 35.3 ±14.3 | 30 days | 35 | 65 | - | - | - |
|  | Commercial | 20 | 33.3 ±11.2 | 30 days | 20 | 80 | - | - | - |
| Tongaonkar et al. | Mosquito net mesh | 359 | - | Up to 5 years | - | - | - | - | - |
|  | Commercial | 60 | - | Up to 5 years | - | - | - | - | - |
| Oribabor et al. | Mosquito net mesh | 130 | 52.27 | Up to 6 months | 58.46 | 41.53 | - | - | - |
| Stephenson et al. | Mosquito net mesh | 54 | 35 | Up to 6 months | - | 87 | - | 3.7 | 9.3 |
| Clarke et al. | Mosquito net mesh | 106 | 44.3 | Up to 6 months | - | 100 |  |  |  |
| Yenli et al. | Mosquito net mesh | 184 | 51 | 1 month | 45 | 55 | - | - | - |
| Rouet et al. | Mosquito net mesh | 41 | 52 | Up to 6 months | - | 82.9 | 17.1 | - | - |

**Table S3** Outcomes: post-operative complications

| **Author** | **Mesh type** | **Overall Complication rate (%)** | **Rejection (%)** | **Recurrence rate (%)** | **Infection (%)** | **Severe or chronic post-operative pain (%)** | **Haematoma or swelling (%)** | **Seroma (%)** | **Impaired wound healing (%)** | **Stitch abscess (%)** | **Other complications (%)** |
| --- | --- | --- | --- | --- | --- | --- | --- | --- | --- | --- | --- |
| Lofgren et al. | Mosquito net mesh | 30.8 | - | 0.7 | 2.8 | 1.4 | 24.5 | 0.7 | 3.5 | - | 1.4 |
|  | Commercial | 29.7 | - | - | 4.1 | - | 23.6 | - | 5.4 | - | 2.7 |
| Chauhan et al. | Indigenous Mesh | 7.5 | - | - | 2.5 | 2.5 | 2.5 | - | - | - | - |
|  | Commercial | 9.1 | - | - | 5 | 2.5 | 2.5 | - | - | - | - |
| Freudenberg et al. | Mosquito net mesh | - | - | - | - | - | - | - | - | - | - |
|  | Commercial | - | - | - | - | - | - | - | - | - | - |
| Tongaonkar et al. | Mosquito net mesh | 7.2 | - | 0.28 | 0.28 | - | 0.84 | 1.12 | - | 4.8 | - |
|  | Commercial | 7.7 | 1.14 | - | 1.14 | - | - | - | - | 6.54 | - |
| Oribabor et al. | Mosquito net mesh | 12.3 | - | - | 4.61 | - | 7.69 | - | - | - | - |
| Stephenson et al. | Mosquito net mesh | - | - | - | - | - | - | - | - | - | - |
| Clarke et al. | Mosquito net mesh | 6.6 | - | - | 1.89 | - | 4.71 | - | - | - | - |
| Yenli et al. | Mosquito net mesh | 10 | - | - | 3 | - | 7 | - | - | - | - |
| Rouet et al. | Mosquiti net mesh | 9.6 | - | - | 2.4 | - | - | 7.2 | - | - | - |
